# Prospective effects of work–time control on overtime, work–life interference and exhaustion in female and male knowledge workers

**DOI:** 10.1177/14034948221150041

**Published:** 2023-02-02

**Authors:** Sophie C. Albrecht, Constanze Leineweber, Göran Kecklund, Philip Tucker

**Affiliations:** 1 Stress Research Institute, Department of Psychology, Stockholm University, Sweden; 2 School of Psychology, Swansea University, UK

**Keywords:** Work–life balance, burnout, long working hours, flexible work, longitudinal

## Abstract

**Aims:**

Employee-based flexible working hours are increasing, particularly among knowledge workers. Research indicates that women and men use work–time control (WTC; control over time off and daily hours) differently: while men work longer paid hours, women use WTC to counteract work–life interference. In a knowledge-worker sample, we examined associations between WTC and overtime, work–life interference and exhaustion and tested whether gender moderates the mediating role of overtime.

**Methods:**

The sample contained 2248 Swedish knowledge workers. Employing hierarchical regression modelling, we examined effects of control over time off/daily hours on subsequent overtime hours, work–life interference and exhaustion in general and in gender-stratified samples. Using conditional process analysis, we tested moderated mediation models.

**Results:**

Control over time off was related to less work–life interference (β_men_= −0.117; 95% confidence interval (CI): −0.237 to 0.003; β_women_= −0.253; 95% CI: −0.386 to −0.120) and lower exhaustion (β_men_= −0.199; 95% CI: −0.347 to −0.051; β_women_= −0.271; 95% CI: −0.443 to −0.100). For control over daily hours, estimates were close to zero. While men worked more overtime (42 min/week), we could not confirm gender moderating the indirect effect of control over time off/daily hours on work–life interference/exhaustion via overtime. Independent of gender, effects of control over time off on work–life interference were partly explained by working fewer overtime hours.

**Conclusions:**

**Control over time off was related to lower exhaustion and better work–life balance (in particular for women). We found no evidence for men’s work–life interference increasing with higher WTC owing to working more overtime. Knowledge workers’ control over time off may help prevent work–life interference and burnout.**

## Background

Flexible working hours are becoming increasingly available to many workers [1], particularly for knowledge-intensive occupations [2], a trend that was accelerated by the coronavirus pandemic [3]. A growing amount of literature highlights one aspect of flexible working hours – work–time control (WTC) – to explain workers’ health and well-being. WTC concerns two sub-dimensions: control over time off (taking breaks, scheduling vacation) and control over daily hours (starting and ending times of work) [4]. A number of studies point towards favourable effects of WTC on exhaustion [5], depressive symptoms [6], work–life interference [7] and even prolonged working life [8]. Some studies found that very high flexibility at work can, however, lead to blurring of boundaries between work and private life [9], which introduces conflicts and stress that are associated with health deterioration [10]. Men in particular seem to be at risk of working more overtime hours with higher control over working hours [11]. Although higher levels of WTC are on average positive for individuals, it is unclear whether and when control can become ‘too much of a good thing’ and if this is the same for women and men. Moreover, the findings of previous studies, that men are more likely than women to use flexibility to facilitate more overtime working, may be flawed because they fail to take the gender-segregated labour market into account – women work more often in occupations with lower degrees of flexibility, whereas men more frequently work in positions with more control over working hours. The present study seeks to address these issues by studying a relatively homogenous sample of Swedish workers within knowledge-intensive services.

Two main mechanisms have been suggested regarding buffering effects of WTC on health. First, WTC hypothetically benefits recovery from work, both within and outside of working hours, by facilitating the taking of breaks when needed and the scheduling of work (in both the short- and the long-term) to allow recovery from strains at work or in private life [12]. Second, WTC evidently buffers against work–life interference by allowing better alignment of work and non-work responsibilities, which in turn is associated with better health outcomes [13]. Although these theories predict that WTC promotes health overall (or prevents ill-health), some findings point towards a difference between men and women in the extent to which they benefit from WTC [14].

An intricate relationship between gender and work–time flexibility has been discussed in research: while women tend to ‘benefit’ from higher levels of WTC, men ‘suffer’ from increased control over working hours [15]. Women seem to use flexibility at work more towards balancing private and work–life needs – for example, by increasing time spent with children [16] and on household tasks [5]. On the other hand, men seem to use flexibility at work more often to further increase working hours, which seems to exacerbate cognitive spill-over and work–life interference [15]. In turn, work-to-home spill-over and conflicts between work and private life are related to a number of negative health and well-being outcomes – for example, exhaustion [17]. This accords with existing research on gender and time use: men tend to work more paid hours while women have a greater total workload including unpaid, domestic labour [18]. Working long hours (i.e. approximately more than 8 h per day/40 h per week) can lead to a number of negative health outcomes, such as stroke, coronary heart disease, depressive mood and sleep problems [19,20].

At the same time, it matters whether overtime hours are worked voluntarily or non-voluntarily; and whether they are compensated or uncompensated. Having control over overtime and receiving rewards for long hours can ameliorate negative effects of overtime on health. Depending on the perceived voluntariness of overtime, long working hours could be related to increased or decreased levels of fatigue and exhaustion [21]. It remains unclear whether higher levels of WTC put men at particular risk of developing burnout syndrome – a condition of emotional exhaustion, physical fatigue and cognitive weariness [22].

Studies suggest that gender differences in the effects of flexible work hours might differ between types of society. While the household burden increased for working mothers in Poland (with more traditional gender roles) when working from home (‘telework’), it did so for both mothers and fathers in Sweden (a relatively egalitarian society) [23]. However, another study found that, even in Sweden, fathers with higher (relative) work flexibility worked more hours at work and reported more time to relax at home, while mothers spent more time on domestic chores [5]. Working more paid hours reduces time available for domestic work and childcare – meaning that WTC may exacerbate gender inequalities and women’s ‘second shift’ of unpaid work. Regardless of society, if men use WTC to increase hours at work, not only would this heighten men’s experience of work–life interference, but would also counteract equal distribution of household and childcare tasks between partners at home.

Studying work- and health-related gender differences is complicated by the issue of gender-segregated labour sectors – horizontally (between industries/occupations) and vertically (between status/power) [24]. Women often work within health care, social work and education, while men predominantly occupy jobs within goods/energy production and machinery operations, thus experiencing very different work conditions – not only in traditional gender-role societies, but also in more egalitarian ones such as Sweden [25]. If this is not taken into consideration, gender comparisons may be biased by occupational gender segregation. Men are often reported to work more overtime than women [11]. At the same time, men report consistently higher levels of WTC than women [4] and may be able to voluntarily increase their working hours more. Women, on the other hand, often work within health/social care and education [25] with fewer opportunities to self-determine working hours. In cross-sector samples, this makes it challenging to investigate gender differences in the effects of overtime and WTC on work–life interference and health: with higher flexibility, do men work more hours at work because they are *men* or because they *can*? And do women with high WTC work more hours if they have the opportunity for overtime work?

One way of addressing these issues is to select a sample from one working sector with approximately equal gender distribution, when studying gender differences. That way, men and women are likely to have similar levels of WTC and opportunities to work overtime. A sector of particular interest here is knowledge-intensive work: with an about-equal distribution of women and men, knowledge work usually entails some degree of flexibility and self-management [26].

In summary, previous research indicates that men with higher levels of WTC tend to work more overtime hours, which in turn is associated with an increase in work–life interference. Women, on the other hand, appear to benefit in terms of work–life balance, but increase total workload/domestic labour. These results may, however, be biased by gender-segregated sectors with differing opportunities to actually self-determine working hours and work overtime. Moreover, in relatively egalitarian societies, women with high WTC potentially work more overtime as well, which in turn could affect both work–life interference and health outcomes.

### Aims

The present study aimed to investigate gender differences in effects of WTC on overtime hours, work–life interference and exhaustion in a sample of Swedish knowledge workers. We examined whether control over time off and daily hours was related to subsequent overtime hours, work–life interference and exhaustion, whether women and men differed in these relations, whether gender moderated effects of WTC on overtime and, last, whether gender moderated the mediating role of overtime in effects of WTC on work–life interference and exhaustion. Additionally, effects of hierarchical position on these relationships were explored in supplementary analyses.

## Methods

### Study design and population

Data came from the Swedish Longitudinal Occupation Survey of Health (SLOSH) – a biennial, postal survey sent to participants of the 2003–2011 Swedish Work Environment Survey (SWES; a sample of the Swedish workforce aged 16–64 years). A full cohort profile can be found elsewhere [27]. The first SLOSH survey was sent out in 2006 to all 2003-SWES respondents; a successively increasing number of participants are followed up every other year. The current study was based on the 2016 and 2018 data collection (response rates 51% and 48%, respectively). In SLOSH, responders are generally more likely to be female, better educated, born in Sweden, older and married or co-habiting than the general working population [27]. Questionnaires differ for those in or outside of paid work (at least 30% of full-time with 100% generally equalling 40 h/week). The present sample included those participating in at least one questionnaire for those in paid work in 2016 (time 1) and 2018 (time 2).

To ensure that any gender differences were not biased by gender-segregated labour sectors, we selected a sample of SLOSH participants in 2016 working within knowledge-intensive services (labelled henceforth as ‘knowledge workers’) – a working domain with a balanced gender distribution and the possibility of both WTC and overtime hours. This information was derived from registry linkage to the Longitudinal Integrated Database for Health Insurance and Labour Market Studies. Occupations were within information and communication, finance and insurance, real estate, professional, scientific and technical activities, including professions such as software engineer, manager, estate agent and accountant [26]. The final sample consisted of *N*=2248 knowledge workers (with 1481 (66%) responding at both time points, 1950 at time 1 and 1779 at time 2). The Regional Research Ethics Board in Stockholm ethically approved both SLOSH and the present study.

### Measures

#### WTC

WTC was measured in 2016 using a five-item scale regarding perceived control over working hours which was rated on a Likert scale from 1 (very little) to 5 (very much) (adapted from Ala-Mursula et al. [28]). Two sub-dimensions can be differentiated [4,28]: *control over time off* (taking breaks, running private errands, scheduling vacation/leave) and *control over daily hours* (starting and ending times of work and length of a workday). Means were calculated per sub-dimension. Cronbach’s alphas were 0.91 for control over time off and 0.80 for control over daily hours.

#### Overtime hours

Hours of overtime were measured in 2016 and 2018 with one item asking ‘How many hours of overtime – paid and unpaid – do you usually work per week?’ and an open answering format. We defined all responses above 30 h as missing [21] as this was deemed unrealistically high and in all likelihood erroneous (*n*=22).

#### Work–life interference

A four-item scale measured work–life interference in 2018 with items such as ‘I come home from work too tired to do things I would like to do’ [29]. Response options were ‘not at all’, ‘rarely’, ‘sometimes’, ‘often’ or ‘almost all the time’ (1–5), which we calculated means for. Cronbach’s alpha was 0.92.

#### Exhaustion

Exhaustion was measured in 2018 using eight items from the Shirom–Melamed-Burnout Questionnaire describing the subscale regarding emotional/physical fatigue [22]. Items were rated on a Likert scale from 1 (almost never) to 7 (almost always), for instance regarding ‘I feel tired’ or ‘My batteries are dead’. Two items were excluded based on lower loadings and previous evidence [30]: ‘I feel refreshed’ and ‘I feel physically exhausted’. Averages were calculated across the remaining six items. Cronbach’s alpha was 0.92.

#### Other variables

Register data were available on participants’ age and gender. Self-reported data at time 1 were used regarding civil status (married/co-habiting or single/alone-living), parental status (no dependable child or at least one child), full-time/part-time work, leading role (differentiating between no leading role or leader/manager with or without subordinates) and occupational skill level based on the Swedish Standard Classification of Occupations (SSYK 2012; [31]; managers/professionals/technicians (higher skill level) versus remaining occupations (lower skills/education)).

### Statistical analysis

Data were prepared with SPSS Statistics, version 27.0 (IBM Corp., Armonk, NY, USA) and analysed using R [32] and the lavaan package [33] as well as the PROCESS macro for SPSS [34]. Descriptive statistics used independent sample *t*-tests. Effects of WTC on overtime hours, work–life interference and exhaustion were tested in separate sequential, multiple regression models utilizing full information maximum likelihood (FIML) to address missing data [35]. Prior to analysing data, we examined underlying assumptions regarding multiple regression, which were met apart from normality of residuals. Due to the Central Limit Theorem and the sample size being sufficiently large, this assumption could be relaxed [36].

**Figure 1.**
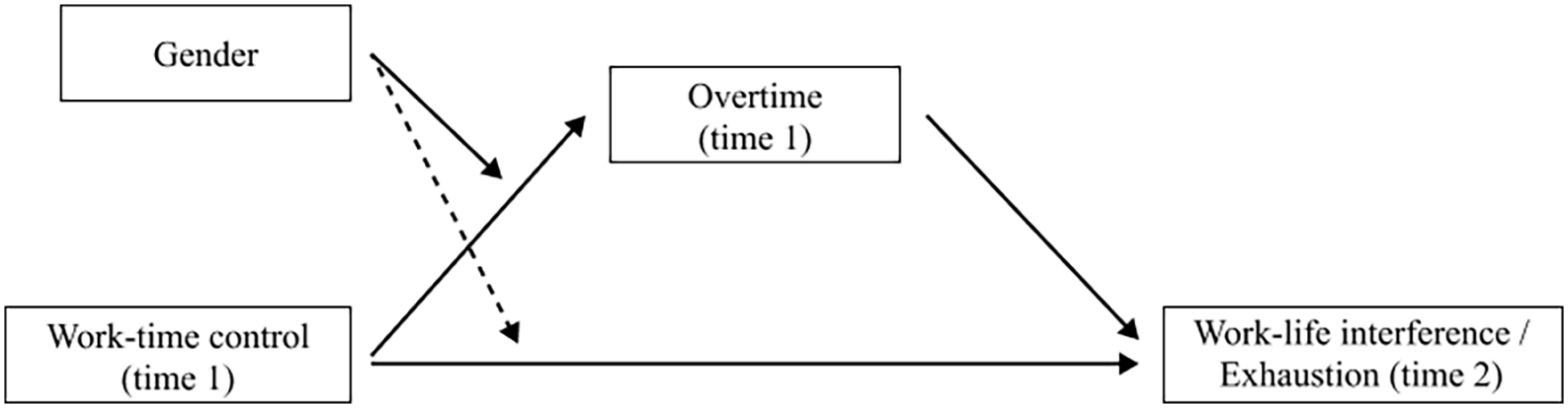
Conceptual diagram of the conditional processes in the analysis on moderated mediation (solid arrow from gender: gender moderating the mediating role of overtime hours between work–time control and work–life interference/exhaustion; dashed arrow from gender: allowing gender to moderate the direct effect of work–time control on work–life interference/exhaustion).

The step-wise analytic procedure was as follows: first, only control over time off and control over daily hours (time 1) were included in regression models regarding effects on overtime, work–life interference or exhaustion (time 2, separate models). Second, we added gender and (only regarding work–life interference and exhaustion) overtime (time 1). Third, we included covariates to the models (age, civil and parental status, managing role, full/part-time work, occupational skill level). We then repeated all analysis steps with gender-stratified samples. In supplementary analyses, these steps were again repeated, differentiating between lower and higher hierarchical position (Supplementary material Tables I to IV online). Last, we tested moderation models regarding overtime (gender moderating effects of WTC (time 1) on overtime (time 2)) and mediated moderation models (gender moderating the mediating role of overtime in effects of either WTC sub-dimension on work–life interference and exhaustion, also allowing gender to moderate direct effects of WTC on work–life interference/exhaustion; see Figure 1) with the PROCESS macro for SPSS (fully adjusted). Confidence intervals (CIs) were bootstrapped based on 10,000 samples.

## Results

### Descriptives

The sample consisted of 1275 male and 973 female knowledge workers (total *N*=2248). Women were on average 49 years (standard deviation (SD)=9), men 51 years (SD=10) old at time 1 at baseline. Sample characteristics can be inspected in Table I. Men reported lower levels of both exhaustion (mean difference (MD)= −0.30, 95% CI −0.43 to −0.18) and work–life interference (MD= −0.11, 95% CI −0.21 to −0.01) than women (all at time 2). In contrast, men reported higher control over time off (MD=0.27, 95% CI 0.20 to 0.35), control over daily hours (MD=0.32, 95% CI 0.22 to 0.42; all at time 1) and number of weekly overtime hours (time 1: MD=0.76, 95% CI 0.35 to 1.18; time 2: MD=1.05, 95% CI 0.62 to 1.47) compared with women.

**Table I.** Characteristics of the sample (at time 1, 2016, unless specified otherwise); higher scores of control over time off/daily hours, work–life interference and exhaustion indicate higher levels/more.

	Men		Women	
	*n*/mean	%/SD	*n*/mean	%/SD
Civil status				
Single/living alone	156	14.4	190	22.3
Married/co-habiting	930	85.6	662	77.7
Parental status				
No children	593	55.0	424	50.4
≧1 child living at home	485	45.0	417	49.6
Working contract				
Full-time	958	90.3	679	83.5
Part-time	103	9.7	134	16.5
Occupational skill level				
Higher skill level	933	88.9	664	79.7
Lower skill level	117	11.1	169	20.3
Leading role				
No leading role	560	53.0	524	64.6
Any leading/managing role	497	47.0	287	35.4
Control over time off (1–5)	4.00	0.78	3.72	0.90
Control over daily hours (1–5)	3.82	1.05	3.50	1.20
Weekly overtime hours (0–30; time 1)	3.80	4.70	3.04	4.17
Weekly overtime hours (0–30; time 2)	3.67	4.66	2.63	3.80
Work–life interference (1–5; time 2)	2.52	0.98	2.63	1.00
Exhaustion (1–7; time 2)	2.17	1.24	2.48	1.36

### Overtime

General and gender-stratified results are displayed in Table II. An increase of 1 unit in control over daily hours (ranging from 1 to 5) was related to, on average, 24 more minutes of overtime at time 2, which in the fully-adjusted model decreased to 12 min and the CI crossed zero. Conversely, control over time off appeared to relate to a decline in overtime hours, with the bulk of the CI being below zero (17 min less per 1-unit increase in control). Men worked on average about 42 min/week more overtime hours than women (fully adjusted results). In gender-stratified analyses, both point estimates regarding control over daily hours and time off were close to zero for women, while men worked on average about 22 min more overtime with a 1-unit increase in control over daily hours and 27 min less overtime with a 1-unit increase in control over time off (CIs crossing zero).

**Table II.** Results from the multiple regression analysis on overtime at time 2 presented overall and stratified by gender.

		Overtime hours − time 2				
			95% confidence interval			
		*B*	Lower	Upper	β	*R* ^2^
	**Time 1**	**General**				
**Model 1**						0.006
	Control over time off	−0.207	−0.623	0.210	−0.040	
	Control over daily hours	0.401	**0.084**	**0.718**	0.104	
**Model 2**						0.019
	Control over time off	−0.245	−0.659	0.169	−0.048	
	Control over daily hours	0.371	**0.056**	**0.686**	0.096	
	Female gender	−0.997	**−1.439**	**−0.555**	−0.114	
**Model 3**						0.083
	Control over time off	−0.279	−0.682	0.124	−0.054	
	Control over daily hours	0.199	−0.114	0.513	0.052	
	Female gender	−0.699	**−1.145**	**−0.253**	−0.080	
	No children living at home	−0.071	−0.582	0.440	−0.008	
	Married/co-habiting	0.169	−0.460	0.799	0.015	
	Age	−0.004	−0.031	0.023	−0.009	
	Lower skill level	−1.007	**−1.721**	**−0.294**	−0.083	
	Any leading role	1.875	**1.385**	**2.366**	0.213	
	Part-time work	−0.715	−1.537	0.106	−0.055	
		**Men**				
**Model 1**						0.010
	Control over time off	−0.456	−1.067	0.155	−0.077	
	Control over daily hours	0.617	**0.149**	**1.084**	0.139	
**Model 2**						0.093
	Control over time off	−0.449	−1.038	0.140	−0.075	
	Control over daily hours	0.370	−0.099	0.839	0.083	
	No children living at home	−0.083	−0.806	0.640	−0.009	
	Married/co-habiting	0.577	−0.414	1.568	0.043	
	Age	−0.018	−0.055	0.020	−0.039	
	Lower skill level	−1.066	−2.249	0.116	−0.072	
	Any leading role	2.255	**1.570**	**2.940**	0.241	
	Part-time work	−1.485	**−2.854**	**−0.116**	−0.094	
		**Women**				
**Model 1**						0.001
	Control over time off	0.008	−0.524	0.540	0.002	
	Control over daily hours	0.097	−0.306	0.499	0.030	
**Model 2**						0.051
	Control over time off	−0.055	−0.580	0.471	−0.013	
	Control over daily hours	0.000	−0.403	0.402	0.000	
	No children living at home	−0.014	−0.706	0.678	−0.002	
	Married/co-habiting	−0.247	−1.015	0.520	−0.027	
	Age	0.015	−0.022	0.051	0.038	
	Lower skill level	−1.096	**−1.928**	**−0.264**	−0.116	
	Any leading role	1.229	**0.543**	**1.916**	0.155	
	Part-time work	−0.156	−1.104	0.792	−0.015	

In the moderation analysis, the interaction terms were −0.278 (95% CI −0.760 to 0.205) between gender and control over daily hours and −0.100 (95% CI −0.735 to 0.535) between gender and control over time off on overtime hours (fully adjusted).

### Work–life interference

Control over time off was related to decreased subsequent work–life interference and remained an important predictor throughout, adding gender, overtime and covariates to the equation (Table III). Point estimates regarding control over daily hours were close to zero. Working overtime and being female both increased the likelihood of perceived work–life interference (albeit zero-crossing CIs in adjusted results). In gender-stratified analyses (Table IV), higher control over time off was related to less work–life interference among women, while point estimates were closer to zero for men. Overtime was associated with an increase in work–life interference similarly for both genders.

**Table III.** Results from the multiple regression analysis regarding work–life interference and exhaustion at time 2 for all knowledge workers.

Time 1	Work–life interference – time 2					Exhaustion – time 2				
		95% confidence interval					95% confidence interval			
	*B*	Lower	Upper	β	*R* ^2^	*B*	Lower	Upper	β	*R* ^2^
**Model 1**					0.028					0.034
Control over time off	−0.204	**−0.297**	**−0.111**	−0.175		−0.230	**−0.344**	**−0.115**	−0.150	
Control over daily hours	0.010	−0.060	0.081	0.012		−0.051	−0.138	0.037	−0.044	
**Model 2**					0.106					0.044
Control over time off	−0.171	**−0.262**	**−0.081**	−0.147		−0.210	**−0.324**	**−0.095**	−0.137	
Control over daily hours	−0.009	−0.077	0.060	−0.010		−0.048	−0.135	0.040	−0.041	
Female gender	0.121	**0.024**	**0.218**	0.060		0.245	**0.122**	**0.368**	0.093	
Overtime hours	0.062	**0.050**	**0.075**	0.281		0.013	−0.003	0.029	0.044	
**Model 3**					0.138					0.091
Control over time off	−0.179	**−0.268**	**−0.090**	−0.153		−0.221	**−0.334**	**−0.109**	−0.145	
Control over daily hours	−0.028	−0.097	0.042	−0.031		−0.043	−0.130	0.044	−0.037	
Female gender	0.086	−0.012	0.184	0.043		0.164	**0.040**	**0.287**	0.062	
Overtime hours	0.062	**0.049**	**0.075**	0.278		0.014	−0.003	0.031	0.049	
No children living at home	−0.050	−0.163	0.063	−0.025		−0.105	−0.251	0.042	−0.040	
Married/co-habiting	−0.214	**−0.352**	**−0.077**	−0.082		−0.213	**−0.388**	**−0.038**	−0.063	
Age	0.012	**0.006**	**0.017**	0.117		0.026	**0.018**	**0.033**	0.199	
Lower skill level	−0.174	**−0.332**	**−0.016**	−0.063		−0.003	−0.193	0.188	−0.001	
Any leading role	0.024	−0.091	0.140	0.012		−0.075	−0.224	0.074	−0.028	
Part-time work	0.148	−0.035	0.330	0.049		0.157	−0.055	0.369	0.040	

**Table IV.** Results from the multiple regression analysis regarding work–life interference and exhaustion at time 2 for male and female knowledge workers.

Time 1	Men					Women				
		95% confidence interval					95% confidence interval			
	*B*	Lower	Upper	β	*R* ^2^	*B*	Lower	Upper	β	*R* ^2^
	**Work – life interference – time 2**									
**Model 1**					0.019					0.037
Control over time off	−0.151	**−0.278**	**−0.024**	−0.120		−0.259	**−0.397**	**−0.122**	−0.235	
Control over daily hours	−0.023	−0.120	0.074	−0.025		0.056	−0.048	0.160	0.068	
**Model 2**					0.107					0.102
Control over time off	−0.107	−0.230	0.015	−0.085		−0.247	**−0.381**	**−0.114**	−0.224	
Control over daily hours	−0.046	−0.140	0.047	−0.049		0.037	−0.064	0.138	0.044	
Overtime hours	0.063	**0.048**	**0.079**	0.301		0.061	**0.040**	**0.081**	0.253	
**Model 3**					0.153					0.138
Control over time off	−0.117	−0.237	0.003	−0.092		−0.253	**−0.386**	**−0.120**	−0.229	
Control over daily hours	−0.073	−0.168	0.023	−0.077		0.026	−0.076	0.129	0.032	
Overtime hours	0.062	**0.046**	**0.078**	0.294		0.062	**0.041**	**0.084**	0.259	
No children living at home	−0.094	−0.242	0.053	−0.047		−0.005	−0.180	0.170	−0.002	
Married/co-habiting	−0.285	**−0.485**	**−0.085**	−0.101		−0.176	−0.367	0.016	−0.073	
Age	0.013	**0.006**	**0.021**	0.135		0.010	**0.001**	**0.019**	0.100	
Lower skill level	−0.133	−0.373	0.107	−0.042		−0.216	**−0.429**	**−0.003**	−0.087	
Any leading role	0.099	−0.048	0.247	0.050		−0.077	−0.263	0.108	−0.037	
Part-time work	0.199	−0.075	0.474	0.060		0.094	−0.154	0.342	0.035	
	**Exhaustion – time 2**									
**Model 1**					0.030					0.028
Control over time off	−0.207	**−0.357**	**−0.056**	−0.131		−0.232	**−0.407**	**−0.057**	−0.154	
Control over daily hours	−0.064	−0.179	0.051	−0.054		−0.020	−0.154	0.114	−0.018	
**Model 2**					0.035					0.028
Control over time off	−0.191	**−0.342**	**−0.040**	−0.121		−0.232	**−0.407**	**−0.056**	−0.154	
Control over daily hours	−0.070	−0.185	0.045	−0.059		−0.020	−0.155	0.114	−0.018	
Overtime hours	0.020	**0.001**	**0.040**	0.078		−0.001	−0.029	0.027	−0.002	
**Model 3**					0.085					0.093
Control over time off	−0.199	**−0.347**	**−0.051**	−0.126		−0.271	**−0.443**	**−0.100**	−0.180	
Control over daily hours	−0.069	−0.184	0.047	−0.058		−0.003	−0.136	0.130	−0.003	
Overtime hours	0.021	**0.001**	**0.041**	0.081		0.004	−0.025	0.033	0.012	
No children living at home	−0.007	−0.195	0.181	−0.003		−0.231	−0.461	0.000	−0.085	
Married/co-habiting	−0.293	**−0.544**	**−0.042**	−0.083		−0.197	−0.443	0.049	−0.060	
Age	0.025	**0.016**	**0.034**	0.208		0.028	**0.017**	**0.040**	0.205	
Lower skill level	0.057	−0.224	0.338	0.014		−0.053	−0.316	0.210	−0.016	
Any leading role	0.062	−0.123	0.248	0.025		−0.264	**−0.508**	**−0.019**	−0.093	
Part-time work	0.336	**0.033**	**0.639**	0.080		−0.013	−0.312	0.286	−0.004	

In the conditional process analysis, CIs were large and crossing zero for interaction terms of control over time off and gender on overtime (0.472, 95% CI −0.134 to 1.078) and on work–life interference (−0.050, 95% CI −0.186 to 0.087). Conditional indirect effects of control over time off on work-life interference via overtime were −0.048 for men (95% CI −0.092 to −0.008) and −0.018 for women (95% CI −0.050 to 0.013). Moderated mediation was not indicated by the index of moderated mediation (0.030, 95% CI −0.014 to 0.079). When testing mediation only, we found non-zero CIs for the indirect effect via overtime (−0.033, 95% CI −0.064 to −0.005); while in the moderation-only model, we failed to confirm gender acting as moderator.

Regarding control over daily hours, unconditional interaction terms with gender were 0.235 (95% CI -−0.229 to 0.700) for effects on overtime and 0.029 (95% CI −0.076 to 0.133) for work–life interference. Conditional indirect effect estimates were close to zero for both women (0.019, 95% CI −0.003 to 0.042) and men (0.004; 95% CI −0.023 to 0.030). From the index of moderated mediation, we could not infer that gender moderates the indirect effect of control over daily hours on work–life interference (0.015, 95% CI −0.016 to 0.046). We explored further models and found neither that overtime alone acted as mediator, nor that gender acted as moderator between control over daily hours and work–life interference.

### Exhaustion

Higher control over time off at time 1 was associated with lower levels of exhaustion at time 2 (Table III). Women were more likely to experience exhaustion. No association between overtime and exhaustion was indicated. In gender-stratified analyses (Table IV), we found that higher levels of control over time off predict lower levels of exhaustion for both men and women, while point estimates regarding overtime were close to zero for both.

In the conditional process analysis, unconditional interaction terms between control over time off and gender were 0.431 (95% CI −0.128 to 0.990) regarding overtime and −0.009 (95% CI −0.157 to 0.174) regarding exhaustion. Estimates of conditional indirect effects were close to zero for women (−0.006; 95% CI −0.020 to 0.002) and men (−0.012; 95% CI to −0.031 to 0.004), as was the index for moderated mediation (0.006, 95% CI −0.006 to 0.022). Neither mediation-only nor moderation-only models produced non-zero CIs regarding interactions/indirect effect estimates.

CIs contained zero for interaction terms between control over daily hours and gender on overtime (0.279, 95% CI −0.149 to 0.707) and exhaustion (0.035, 95% CI −0.092 to 0.162). Conditional indirect effect estimates were close to zero for both women (0.004, 95% CI −0.001 to 0.014) and men (0.001, 95% CI −0.005 to 0.008), as was the index for moderated mediation (0.004, 95% CI −0.004 to 0.015). Again, mediation-only and moderation-only models produced CIs containing zero regarding interactions/indirect effect estimates.

## Discussion

In this prospective study of knowledge workers from the Swedish SLOSH cohort, higher control over time off was associated with a decrease in work–life interference and exhaustion, while associations with control over daily hours were close to zero. We found no evidence that the indirect effect of WTC on work–life interference/exhaustion via overtime was dependent on gender. The latter finding stands in contrast to previous studies indicating that men tend to use higher WTC to increase their paid working hours, thereby exacerbating work–life interference, while women use the flexibility to counteract work–life interference [5,11,14].

### Control over time off versus control over daily hours

Although often disregarded in research, WTC is a two-dimensional construct comprising control over daily hours (starting/ending times) and control over time off (breaks, vacation, leave of absence) [4,28]. These sub-dimensions seem to differ in terms of what control workers have/need/use [37] and how they relate to health [13]. In our results, control over time off was associated more consistently with less work–life interference and exhaustion than control over daily hours. Moreover, the sub-dimensions were related to overtime in opposite directions (albeit mostly with zero-crossing CIs), particularly for men, suggesting that a one-dimensional measure of WTC may in fact mask any differential effects. For example, while a German study found that higher WTC predicted *increased* overtime among both men and women [11], we found those with higher control over time off worked fewer overtime hours and in turn perceived less work–life interference (independent of gender). This contrast in findings illustrates how the different aspects of WTC can have dissimilar effects.

### Gender-segregated labour sectors

In contrast to previous research, we used a sample restricted to knowledge workers, where women and men have similar opportunities for control over working hours as well as working overtime. Thus, we minimized the possibility that observed relationships between WTC, overtime and work–life interference are explained by women and men working in different occupations and consequently in different work environments. Our results highlight that previous findings on gender differences in the effects of flexible working hours on work–life balance may be at least partially confounded by gender-segregation between occupational sectors. Employees in health/social care and education – the majority of them being women [25] – less commonly have the opportunity to self-determine working hours (other than in a very different format, such as through participatory working time/day scheduling within shift work) [24].

### Gender differences

While the absence of gender-moderated effects may suggest a positive picture of Sweden’s gender equality, we still found notable gender differences. Men worked more overtime hours than women. As working more leaves less time for domestic labour/childcare [14] and relaxation, this finding adds to previous evidence of Swedish women and men using unequal amounts of time for paid and unpaid work, children and even leisure [5,38]. Men’s overtime tended to decrease with higher control over time off and increase with higher control over daily hours, yet women appeared to benefit more from control over time off in terms of work–life interference. Nevertheless, while we find some evidence for traditional gender-role time allocation, control over time off appears not to further enhance gender inequalities in health and wellbeing. Rather, it helps in buffering against exhaustion and work–life interference among both female and male knowledge workers.

It is notable that holding a managing position was associated with more overtime hours for both genders. Even in an egalitarian society like Sweden, men are more likely than women to occupy senior positions at work [24]. It seems likely that this gender-biased selection into hierarchical positions, as well as gender biases in other work-related characteristics, will play a role in the observed gender differences in time allocation. If control over daily hours does indeed lead to more hours of overtime for men, but not women, this could even contribute to vertical segregation by gender. Future research should examine the importance of seniority versus gender in the relationships between WTC, overtime, work–life balance and health.

### Limitations

Sample participants worked about 3 h of weekly overtime on average, which may have been too low to observe stronger effects on work–life interference and exhaustion, particularly as we included part-time workers in the sample. However, other studies that defined anything above 8 h/day or 40 h/week as long working hours still found that longer hours produced effects on major health outcomes [20]. In a similar vein, low frequency of low levels of control may have contributed to underestimated effect estimates regarding effects on work–life interference/exhaustion. While this study focuses on horizontal segregation by gender, vertical segregation is considered only as covariate, which could introduce bias if women and men in the knowledge working sector are unequally distributed in levels of hierarchy. Although lower in power, results from the supplementary analysis (Supplementary Tables I to V) grouping female and male knowledge workers into those in lower and higher hierarchical positions point to no major differences from the main results.

Although we address a number of potential confounders, we cannot rule out bias due to unmeasured confounding, including unobserved heterogeneity. While SLOSH is based on a broadly representative sample of the Swedish working population, (self-) selection processes of participants are still likely to be present, particularly for those responding several times, which may introduce selection bias. Although the study design is prospective, variables were not analysed using repeated measurements over time. Likewise, attrition and missing data may have added a degree of bias to our results; we tried to limit this by utilizing FIML for data missing at random [35]. Data used are mostly self-reported, which comes with the downside of common-method bias and measurement error. The scales to measure relevant constructs are well-established, include multiple items and have good internal consistency, which indicates lower likelihood of high random and systematic measurement errors. Finally, the present sample concerns knowledge workers only and specific recommendations are to some degree limited in their generalizability to other sectors.

## Conclusions

Results presented here give a nuanced picture of gender-specific use of flexible working hours and effects on overtime hours, work–life interference and exhaustion in a sample of Swedish knowledge workers. Control over time off stood out as being particularly beneficial. More control over time off was related to less work–life interference (particularly for women) and lower exhaustion. Based on our results, employers seeking to improve male or female knowledge workers’ work–life balance and counteract feelings of exhaustion and burnout should focus on increasing individual control over when not to work (taking breaks, running private errands during work and scheduling vacation/other leave) while at the same time preventing employees from further increasing their working hours.

## Supplemental Material

sj-docx-1-sjp-10.1177_14034948221150041 – Supplemental material for Prospective effects of work–time control on overtime, work–life interference and exhaustion in female and male knowledge workers Supplemental material, sj-docx-1-sjp-10.1177_14034948221150041 for Prospective effects of work–time control on overtime, work–life interference and exhaustion in female and male knowledge workers by Sophie C. Albrecht, Constanze Leineweber, Göran Kecklund and Philip Tucker in Scandinavian Journal of Public Health
