# Supplementary material for: Prospective effects of work–time control on overtime, work–life interference and exhaustion in female and male knowledge workers
Source: Scand J Public Health. 2023 Feb 2;52(2):205–15. doi: 10.1177/14034948221150041 (PMC10913321; doi:10.1177/14034948221150041)
Supplement: sj-docx-1-sjp-10.1177_14034948221150041 – Supplemental material for Prospective effects of work–time control on overtime, work–life interference and exhaustion in female and male knowledge workers [file sj-docx-1-sjp-10.1177_14034948221150041.docx]

Supplemental Table 1: Results from the multiple regression analysis on overtime at time 2 presented overall and stratified by gender and separated between those holding no leading role (lower hierarchical position) and any leading role (higher hierarchical position).

|  |  | **Overtime hours - Time 2** | | | | | | | | | | | | | | | | | | | | | | | | | | | | |  |
| --- | --- | --- | --- | --- | --- | --- | --- | --- | --- | --- | --- | --- | --- | --- | --- | --- | --- | --- | --- | --- | --- | --- | --- | --- | --- | --- | --- | --- | --- | --- | --- |
|  |  |  | | | **Confidence interval 95%** | | | | | | |  | | |  | | |  | | | **Confidence interval 95%** | | | | | | |  |  | |  |
|  |  | **B** | | | **lower** | | | | **upper** | | | **beta** | | | **R2** | | | **B** | | | **lower** | | | | **upper** | | | **beta** | **R2** | |  |
|  | **Time 1** | **Lower hierarchical position** | | | | | | | | | | | | | | | | | **Higher hierarchical position** | | | | | | | | | | | |  |
| **Model 1** | |  |  | | |  | | | |  | | |  | | |  | | | |  | | |  | | |  | | | 0.005 | |  |
|  | Control over time off | 0.005 | -0.450 | | | 0.460 | | | | 0.001 | | |  | | | -0.609 | | | | -1.383 | | | 0.164 | | | -0.098 | | |  | |  |
|  | Control over daily hours | 0.271 | -0.060 | | | 0.601 | | | | 0.089 | | |  | | | 0.225 | | | | -0.421 | | | 0.870 | | | 0.044 | | |  | |  |
| **Model 2** | |  |  | | |  | | | |  | | | 0.010 | | |  | | | |  | | |  | | |  | | | 0.024 | |  |
|  | Control over time off | -0.014 | -0.470 | | | 0.441 | | | | -0.003 | | |  | | | -0.657 | | | | -1.423 | | | 0.110 | | | -0.106 | | |  | |  |
|  | Control over daily hours | 0.267 | -0.063 | | | 0.597 | | | | 0.088 | | |  | | | 0.162 | | | | -0.478 | | | 0.802 | | | 0.032 | | |  | |  |
|  | Female gender | -0.328 | -0.881 | | | 0.225 | | | | -0.045 | | |  | | | -1.439 | | | | **-2.314** | | | **-0.564** | | | -0.141 | | |  | |  |
| **Model 3** | |  |  | | |  | | | |  | | | 0.031 | | |  | | | |  | | |  | | |  | | |  | |  |
|  | Control over time off | 0.012 | -0.441 | | | 0.466 | | | | 0.003 | | |  | | | -0.696 | | | | -1.467 | | | 0.074 | | | -0.112 | | |  | |  |
|  | Control over daily hours | 0.205 | -0.129 | | | 0.539 | | | | 0.067 | | |  | | | 0.117 | | | | -0.539 | | | 0.773 | | | 0.023 | | |  | |  |
|  | Female gender | -0.200 | -0.760 | | | 0.360 | | | | -0.027 | | |  | | | -1.208 | | | | **-2.110** | | | **-0.306** | | | -0.119 | | |  | |  |
|  | No children living at home | -0.139 | -0.740 | | | 0.462 | | | | -0.019 | | |  | | | -0.075 | | | | -0.997 | | | 0.847 | | | -0.007 | | |  | |  |
|  | Married/co-habiting | -0.002 | -0.726 | | | 0.722 | | | | 0.000 | | |  | | | 0.521 | | | | -0.664 | | | 1.705 | | | 0.039 | | |  | |  |
|  | Age | 0.011 | -0.021 | | | 0.042 | | | | 0.030 | | |  | | | -0.027 | | | | -0.077 | | | 0.024 | | | -0.051 | | |  | |  |
|  | Lower skill level | -1.053 | -1.776 | | | -0.330 | | | | -0.118 | | |  | | | -1.015 | | | | -2.959 | | | 0.930 | | | -0.047 | | |  | |  |
|  | Part-time work | -0.449 | -1.333 | | | 0.435 | | | | -0.046 | | |  | | | -1.740 | | | | -3.489 | | | 0.009 | | | -0.093 | | |  | |  |
|  |  |  | | |  | | |  | | | |  | | |  | | |  | | |  | | | |  | | |  |  | |  |
|  |  | **Men**  **Lower hierarchical position** | | | | | | | | | | | | | | | | **Women**  **Lower hierarchical position** | | | | | | | | | | | | |  |
| **Model 1** | |  |  | | |  | | | |  | | | 0.018 | | |  | | | |  | | |  | | |  | | | 0.001 | |  |
|  | Control over time off | -0.103 | -0.775 | | | 0.569 | | | | -0.022 | | |  | | | 0.098 | | | | -0.518 | | | 0.713 | | |  | | |  | |  |
|  | Control over daily hours | 0.481 | **0.009** | | | **0.952** | | | | 0.147 | | |  | | | 0.038 | | | | -0.422 | | | 0.498 | | |  | | |  | |  |
| **Model 2** | |  |  | | |  | | | |  | | | 0.024 | | |  | | | |  | | |  | | |  | | | 0.044 | |  |
|  | Control over time off | -0.077 | -0.749 | | | 0.596 | | | | -0.016 | | |  | | | 0.105 | | | | -0.504 | | | 0.714 | | | 0.027 | | |  | |  |
|  | Control over daily hours | 0.421 | -0.065 | | | 0.907 | | | | 0.128 | | |  | | | -0.006 | | | | -0.467 | | | 0.454 | | | -0.002 | | |  | |  |
|  | No children living at home | 0.008 | -0.838 | | | 0.854 | | | | 0.001 | | |  | | | -0.232 | | | | -1.087 | | | 0.623 | | | -0.033 | | |  | |  |
|  | Married/co-habiting | -0.011 | -1.107 | | | 1.085 | | | | -0.001 | | |  | | | -0.021 | | | | -0.978 | | | 0.936 | | | -0.003 | | |  | |  |
|  | Age | -0.003 | -0.046 | | | 0.040 | | | | -0.009 | | |  | | | 0.031 | | | | -0.015 | | | 0.078 | | | 0.089 | | |  | |  |
|  | Lower skill level | -0.663 | -1.846 | | | 0.519 | | | | -0.065 | | |  | | | -1.267 | | | | **-2.172** | | | **-0.361** | | | -0.159 | | |  | |  |
|  | Part-time work | -0.680 | -2.153 | | | 0.794 | | | | -0.060 | | |  | | | -0.186 | | | | -1.275 | | | 0.904 | | | -0.021 | | |  | |  |
|  |  |  |  | | |  | | | |  | | |  | | |  | | | |  | | |  | | |  | | |  | |  |
|  |  |  |  | | |  | | | |  | | |  | | |  | | | |  | | |  | | |  | | |  | |  |
|  |  |  | | |  | | | |  | | |  | | |  | | |  | | |  | | | |  | | |  |  | |  |
|  |  | **Men**  **Higher hierarchical position** | | | | | | | | | | | | | | | | **Women**  **Higher hierarchical position** | | | | | | | | | | | | |  |
| **Model 1** | |  | |  | | |  | | | |  | | | 0.001 | | |  | | | | |  | |  | | |  | | | 0.002 |  |
|  | Control over time off | -0.947 | | -1.984 | | | 0.091 | | | | -0.133 | | |  | | | -0.148 | | | | | -1.228 | | 0.933 | | | -0.030 | | |  |  |
|  | Control over daily hours | 0.271 | | -0.649 | | | 1.191 | | | | 0.046 | | |  | | | -0.058 | | | | | -0.895 | | 0.780 | | | -0.015 | | |  |  |
| **Model 2** | |  | |  | | |  | | | |  | | | 0.045 | | |  | | | | |  | |  | | |  | | | 0.012 |  |
|  | Control over time off | -0.919 | | -1.959 | | | 0.120 | | | | -0.129 | | |  | | | -0.135 | | | | | -1.267 | | 0.998 | | | -0.028 | | |  |  |
|  | Control over daily hours | 0.294 | | -0.659 | | | 1.247 | | | | 0.050 | | |  | | | -0.105 | | | | | -0.963 | | 0.753 | | | -0.027 | | |  |  |
|  | No children living at home | -0.189 | | -1.432 | | | 1.054 | | | | -0.017 | | |  | | | 0.227 | | | | | -1.080 | | 1.533 | | | 0.027 | | |  |  |
|  | Married/co-habiting | 1.833 | | -0.062 | | | 3.729 | | | | 0.109 | | |  | | | -0.520 | | | | | -1.907 | | 0.868 | | | -0.055 | | |  |  |
|  | Age | -0.042 | | -0.109 | | | 0.025 | | | | -0.077 | | |  | | | -0.016 | | | | | -0.091 | | 0.059 | | | -0.034 | | |  |  |
|  | Lower skill level | -1.019 | | -3.982 | | | 1.944 | | | | -0.041 | | |  | | | -1.242 | | | | | -3.606 | | 1.121 | | | -0.076 | | |  |  |
|  | Part-time work | -2.962 | | **-5.655** | | | **-0.270** | | | | -0.138 | | |  | | | -0.126 | | | | | -2.303 | | 2.051 | | | -0.009 | | |  |  |
|  |  |  |  | | |  | | | |  | | |  | | |  | | | |  | | |  | | |  | | |  | |  |

Supplemental Table 2: Results from the multiple regression analysis regarding work–life interference at Time 2 for all knowledge workers separated between those holding no leading role (lower hierarchical position) and any leading role (higher hierarchical position).

|  |  | **Work–Life Interference - Time 2** | | | | | | | | | | | | | | | | | |  |
| --- | --- | --- | --- | --- | --- | --- | --- | --- | --- | --- | --- | --- | --- | --- | --- | --- | --- | --- | --- | --- |
|  |  |  | **Confidence interval 95%** | | |  | | |  |  |  | | | **Confidence interval 95%** | |  | |  | |  |
|  | **Time 1** | **B** | | **lower** | **upper** | | **beta** | **R^2^** | |  | | **B** | **lower** | | **upper** | | **beta** | | **R^2^** | |
|  | | **Lower hierarchical position** | | | | | | | |  | | **Higher hierarchical position** | | | | | | | | |
| **Model 1** | |  | |  |  | |  | 0.043 | |  | |  |  | |  | |  | | 0.022 | |
|  | Control over time off | -0.218 | | **-0.334** | **-0.102** | | -0.194 |  | |  | | -0.216 | **-0.367** | | **-0.065** | | -0.172 | |  | |
|  | Control over daily hours | -0.014 | | -0.098 | 0.070 | | -0.018 |  | |  | | 0.036 | -0.090 | | 0.162 | | 0.035 | |  | |
| **Model 2** | |  | |  |  | |  | 0.113 | |  | |  |  | |  | |  | | 0.101 | |
|  | Control over time off | -0.195 | | **-0.308** | **-0.082** | | -0.172 |  | |  | | -0.170 | **-0.317** | | **-0.024** | | -0.135 | |  | |
|  | Control over daily hours | -0.032 | | -0.114 | 0.050 | | -0.040 |  | |  | | 0.044 | -0.077 | | 0.166 | | 0.043 | |  | |
|  | Female gender | 0.189 | | **0.050** | **0.328** | | 0.097 |  | |  | | 0.088 | -0.078 | | 0.255 | | 0.043 | |  | |
|  | Overtime hours | 0.073 | | **0.050** | **0.096** | | 0.253 |  | |  | | 0.054 | **0.038** | | **0.071** | | 0.284 | |  | |
| **Model 3** | |  | |  |  | |  | 0.153 | |  | |  |  | |  | |  | |  | |
|  | Control over time off | -0.203 | | **-0.314** | **-0.091** | | -0.179 |  | |  | | -0.186 | **-0.332** | | **-0.040** | | -0.147 | |  | |
|  | Control over daily hours | -0.057 | | -0.140 | 0.025 | | -0.070 |  | |  | | 0.071 | -0.054 | | 0.195 | | 0.068 | |  | |
|  | Female gender | 0.178 | | **0.039** | **0.316** | | 0.091 |  | |  | | 0.010 | -0.160 | | 0.180 | | 0.005 | |  | |
|  | Overtime hours | 0.072 | | **0.050** | **0.095** | | 0.249 |  | |  | | 0.056 | **0.040** | | **0.072** | | 0.292 | |  | |
|  | No children living at home | -0.085 | | -0.234 | 0.065 | | -0.043 |  | |  | | 0.021 | -0.155 | | 0.196 | | 0.010 | |  | |
|  | Married/co-habiting | -0.164 | | -0.342 | 0.015 | | -0.066 |  | |  | | -0.305 | **-0.531** | | **-0.079** | | -0.113 | |  | |
|  | Age | 0.011 | | **0.004** | **0.019** | | 0.119 |  | |  | | 0.012 | **0.003** | | **0.022** | | 0.114 | |  | |
|  | Lower skill level | -0.240 | | **-0.420** | **-0.060** | | -0.100 |  | |  | | 0.096 | -0.269 | | 0.461 | | 0.022 | |  | |
|  | Part-time work | 0.204 | | -0.015 | 0.423 | | 0.077 |  | |  | | -0.067 | -0.394 | | 0.260 | | -0.018 | |  | |
|  |  |  | |  |  | |  |  | |  | |  |  | |  | |  | |  | |

Supplemental Table 3: Results from the multiple regression analysis regarding exhaustion at Time 2 for all knowledge workers separated between those holding no leading role (lower hierarchical position) and any leading role (higher hierarchical position).

|  |  | **Exhaustion - Time 2** | | | | | | | | | | | | | | | | | |  |
| --- | --- | --- | --- | --- | --- | --- | --- | --- | --- | --- | --- | --- | --- | --- | --- | --- | --- | --- | --- | --- |
|  |  |  | **Confidence interval 95%** | | |  | | |  |  |  | | | **Confidence interval 95%** | |  | |  | |  |
|  | **Time 1** | **B** | | **lower** | **upper** | | **beta** | **R^2^** | |  | | **B** | **lower** | | **upper** | | **beta** | | **R^2^** | |
|  | | **Lower hierarchical position** | | | | | | | |  | | **Higher hierarchical position** | | | | | | | | |
| **Model 1** | |  | |  |  | |  | 0.031 | |  | |  |  | |  | |  | | 0.033 | |
|  | Control over time off | -0.200 | | **-0.342** | **-0.058** | | -0.132 |  | |  | | -0.280 | **-0.458** | | **-0.101** | | -0.181 | |  | |
|  | Control over daily hours | -0.060 | | -0.164 | 0.044 | | -0.055 |  | |  | | -0.003 | -0.152 | | 0.145 | | -0.003 | |  | |
| **Model 2** | |  | |  |  | |  | 0.045 | |  | |  |  | |  | |  | | 0.039 | |
|  | Control over time off | -0.181 | | **-0.322** | **-0.039** | | -0.119 |  | |  | | -0.257 | **-0.437** | | **-0.078** | | -0.167 | |  | |
|  | Control over daily hours | -0.056 | | -0.160 | 0.047 | | -0.052 |  | |  | | 0.000 | -0.148 | | 0.148 | | 0.000 | |  | |
|  | Female gender | 0.310 | | **0.134** | **0.487** | | 0.119 |  | |  | | 0.117 | -0.088 | | 0.322 | | 0.046 | |  | |
|  | Overtime hours | 0.011 | | -0.017 | 0.038 | | 0.028 |  | |  | | 0.016 | -0.004 | | 0.036 | | 0.067 | |  | |
| **Model 3** | |  | |  |  | |  | 0.096 | |  | |  |  | |  | |  | | 0.083 | |
|  | Control over time off | -0.200 | | **-0.339** | **-0.062** | | -0.132 |  | |  | | -0.257 | **-0.435** | | **-0.080** | | -0.167 | |  | |
|  | Control over daily hours | -0.054 | | -0.158 | 0.049 | | -0.050 |  | |  | | 0.023 | -0.127 | | 0.172 | | 0.018 | |  | |
|  | Female gender | 0.261 | | **0.087** | **0.436** | | 0.100 |  | |  | | -0.012 | -0.219 | | 0.195 | | -0.005 | |  | |
|  | Overtime hours | 0.009 | | -0.018 | 0.036 | | 0.022 |  | |  | | 0.019 | -0.001 | | 0.038 | | 0.079 | |  | |
|  | No children living at home | 0.022 | | -0.172 | 0.216 | | 0.008 |  | |  | | -0.179 | -0.393 | | 0.036 | | -0.072 | |  | |
|  | Married/co-habiting | -0.075 | | -0.299 | 0.150 | | -0.022 |  | |  | | -0.430 | **-0.706** | | **-0.154** | | -0.130 | |  | |
|  | Age | 0.029 | | **0.019** | **0.038** | | 0.227 |  | |  | | 0.019 | **0.008** | | **0.031** | | 0.149 | |  | |
|  | Lower skill level | -0.035 | | -0.252 | 0.181 | | -0.011 |  | |  | | 0.114 | -0.324 | | 0.551 | | 0.021 | |  | |
|  | Part-time work | 0.129 | | -0.118 | 0.377 | | 0.037 |  | |  | | 0.108 | -0.272 | | 0.488 | | 0.023 | |  | |
|  |  |  | |  |  | |  |  | |  | |  |  | |  | |  | |  | |

Supplemental Table 4: Results from the multiple regression analysis regarding work–life interference at time 2 for **male** and **female** knowledge workers separated between those holding no leading role (lower hierarchical position) and any leading role (higher hierarchical position).

|  |  | **Work–life interference - Time 2** | | | | | | | | | | |  |
| --- | --- | --- | --- | --- | --- | --- | --- | --- | --- | --- | --- | --- | --- |
|  |  | **Men** | | | | |  | **Women** | | | | |  |
|  |  |  | **Confidence Interval 95%** | |  |  |  |  | **Confidence Interval 95%** | |  |  |  |
|  | **Time 1** | **B** | **lower** | **upper** | **beta** | **R2** |  | **B** | **lower** | **upper** | **beta** | **R2** |  |
|  | | **Lower hierarchical position** | | | | |  | **Lower hierarchical position** | | | | |  |
| **Model 1** | |  |  |  |  | 0.053 |  |  |  |  |  | 0.031 |  |
|  | Control over time off | -0.199 | **-0.365** | **-0.032** | -0.167 |  |  | -0.230 | **-0.392** | **-0.068** | -0.214 |  |  |
|  | Control over daily hours | -0.068 | -0.185 | 0.048 | -0.082 |  |  | 0.046 | -0.074 | 0.166 | 0.058 |  |  |
| **Model 2** | |  |  |  |  | 0.135 |  |  |  |  |  | 0.083 |  |
|  | Control over time off | -0.162 | -0.324 | 0.000 | -0.135 |  |  | -0.234 | **-0.392** | **-0.075** | -0.216 |  |  |
|  | Control over daily hours | -0.104 | -0.218 | 0.010 | -0.124 |  |  | 0.040 | -0.078 | 0.157 | 0.050 |  |  |
|  | Overtime hours | 0.071 | **0.043** | **0.099** | 0.279 |  |  | 0.078 | **0.039** | **0.116** | 0.228 |  |  |
| **Model 3** | |  |  |  |  | 0.200 |  |  |  |  |  | 0.115 |  |
|  | Control over time off | -0.177 | **-0.334** | **-0.020** | -0.146 |  |  | -0.245 | **-0.402** | **-0.087** | -0.226 |  |  |
|  | Control over daily hours | -0.134 | **-0.248** | **-0.020** | -0.158 |  |  | 0.025 | -0.094 | 0.143 | 0.031 |  |  |
|  | Overtime hours | 0.073 | **0.046** | **0.101** | 0.285 |  |  | 0.075 | **0.036** | **0.113** | 0.218 |  |  |
|  | No children living at home | -0.243 | **-0.440** | **-0.045** | -0.125 |  |  | 0.088 | -0.138 | 0.313 | 0.044 |  |  |
|  | Married/co-habiting | -0.151 | -0.405 | 0.103 | -0.058 |  |  | -0.184 | -0.434 | 0.066 | -0.077 |  |  |
|  | Age | 0.012 | **0.002** | **0.022** | 0.134 |  |  | 0.010 | -0.002 | 0.023 | 0.107 |  |  |
|  | Lower skill level | -0.250 | -0.528 | 0.028 | -0.095 |  |  | -0.246 | **-0.485** | **-0.006** | -0.111 |  |  |
|  | Part-time work | 0.338 | -0.005 | 0.680 | 0.115 |  |  | 0.102 | -0.186 | 0.391 | 0.042 |  |  |
|  | | **Higher hierarchical position** | | | | |  | **Higher hierarchical position** | | | | |  |
| **Model 1** | |  |  |  |  | 0.008 |  |  |  |  |  | 0.056 |  |
|  | Control over time off | -0.150 | -0.341 | 0.042 | -0.111 |  |  | -0.324 | **-0.568** | **-0.080** | -0.281 |  |  |
|  | Control over daily hours | 0.043 | -0.125 | 0.211 | 0.038 |  |  | 0.055 | -0.136 | 0.247 | 0.060 |  |  |
| **Model 2** | |  |  |  |  | 0.096 |  |  |  |  |  | 0.131 |  |
|  | Control over time off | -0.102 | -0.286 | 0.082 | -0.075 |  |  | -0.287 | **-0.525** | **-0.049** | -0.247 |  |  |
|  | Control over daily hours | 0.075 | -0.086 | 0.237 | 0.067 |  |  | 0.038 | -0.148 | 0.224 | 0.041 |  |  |
|  | Overtime hours | 0.059 | **0.038** | **0.080** | 0.305 |  |  | 0.049 | **0.023** | **0.076** | 0.262 |  |  |
| **Model 3** | |  |  |  |  | 0.161 |  |  |  |  |  | 0.145 |  |
|  | Control over time off | -0.105 | -0.288 | 0.078 | -0.077 |  |  | -0.319 | **-0.567** | **-0.072** | -0.275 |  |  |
|  | Control over daily hours | 0.108 | -0.058 | 0.275 | 0.096 |  |  | 0.062 | -0.128 | 0.252 | 0.067 |  |  |
|  | Overtime hours | 0.063 | **0.042** | **0.083** | 0.323 |  |  | 0.051 | **0.024** | **0.077** | 0.269 |  |  |
|  | No children living at home | 0.124 | -0.096 | 0.345 | 0.060 |  |  | -0.152 | -0.436 | 0.133 | -0.075 |  |  |
|  | Married/co-habiting | -0.539 | **-0.876** | **-0.201** | -0.168 |  |  | -0.142 | -0.443 | 0.159 | -0.062 |  |  |
|  | Age | 0.015 | **0.003** | **0.027** | 0.147 |  |  | 0.006 | -0.010 | 0.023 | 0.053 |  |  |
|  | Lower skill level | 0.154 | -0.341 | 0.650 | 0.032 |  |  | 0.106 | -0.428 | 0.640 | 0.027 |  |  |
|  | Part-time work | -0.197 | -0.650 | 0.256 | -0.048 |  |  | -0.078 | -0.564 | 0.407 | -0.023 |  |  |

Supplemental Table 5: Results from the multiple regression analysis regarding exhaustion at time 2 for **male** and **female** knowledge workers separated between those holding no leading role (lower hierarchical position) and any leading role (higher hierarchical position).

|  |  | **Exhaustion - Time 2** | | | | | | | | | | |  |
| --- | --- | --- | --- | --- | --- | --- | --- | --- | --- | --- | --- | --- | --- |
|  |  | **Men** | | | | |  | **Women** | | | | |  |
|  |  |  | **Confidence Interval 95%** | |  |  |  |  | **Confidence Interval 95%** | |  |  |  |
|  | **Time 1** | **B** | **lower** | **upper** | **beta** | **R2** |  | **B** | **lower** | **upper** | **beta** | **R2** |  |
|  |  |  | | | | | | | | | | | |
|  | | **Lower hierarchical position** | | | | |  | **Lower hierarchical position** | | | | |  |
| **Model 1** | |  |  |  |  | 0.059 |  |  |  |  |  | 0.009 |  |
|  | Control over time off | -0.254 | **-0.439** | **-0.069** | -0.171 |  |  | -0.134 | -0.348 | 0.079 | -0.088 |  |  |
|  | Control over daily hours | -0.097 | -0.229 | 0.035 | -0.093 |  |  | -0.009 | -0.169 | 0.151 | -0.008 |  |  |
| **Model 2** | |  |  |  |  | 0.063 |  |  |  |  |  | 0.010 |  |
|  | Control over time off | -0.239 | **-0.425** | **-0.053** | -0.160 |  |  | -0.135 | -0.348 | 0.079 | -0.088 |  |  |
|  | Control over daily hours | -0.106 | -0.238 | 0.026 | -0.101 |  |  | -0.007 | -0.167 | 0.153 | -0.006 |  |  |
|  | Overtime hours | 0.020 | -0.010 | 0.050 | 0.064 |  |  | -0.015 | -0.069 | 0.039 | -0.031 |  |  |
| **Model 3** | |  |  |  |  | 0.125 |  |  |  |  |  | 0.070 |  |
|  | Control over time off | -0.245 | **-0.425** | **-0.064** | -0.164 |  |  | -0.167 | -0.376 | 0.042 | -0.109 |  |  |
|  | Control over daily hours | -0.096 | -0.228 | 0.035 | -0.092 |  |  | -0.005 | -0.164 | 0.153 | -0.005 |  |  |
|  | Overtime hours | 0.022 | -0.007 | 0.052 | 0.070 |  |  | -0.034 | -0.087 | 0.020 | -0.069 |  |  |
|  | No children living at home | 0.121 | -0.119 | 0.361 | 0.050 |  |  | -0.116 | -0.423 | 0.191 | -0.042 |  |  |
|  | Married/co-habiting | -0.130 | -0.433 | 0.172 | -0.041 |  |  | -0.057 | -0.385 | 0.271 | -0.017 |  |  |
|  | Age | 0.028 | **0.017** | **0.040** | 0.251 |  |  | 0.029 | **0.014** | **0.045** | 0.211 |  |  |
|  | Lower skill level | 0.061 | -0.245 | 0.368 | 0.019 |  |  | -0.136 | -0.443 | 0.172 | -0.043 |  |  |
|  | Part-time work | 0.370 | **0.032** | **0.709** | 0.102 |  |  | -0.088 | -0.446 | 0.271 | -0.025 |  |  |
|  |  |  |  |  |  |  |  |  |  |  |  |  |  |
|  | | **Higher hierarchical position** | | | | |  | **Higher hierarchical position** | | | | |  |
| **Model 1** | |  |  |  |  | 0.009 |  |  |  |  |  | 0.084 |  |
|  | Control over time off | -0.176 | -0.404 | 0.053 | -0.107 |  |  | -0.419 | **-0.705** | **-0.133** | -0.291 |  |  |
|  | Control over daily hours | 0.021 | -0.173 | 0.215 | 0.015 |  |  | 0.002 | -0.228 | 0.232 | 0.002 |  |  |
| **Model 2** | |  |  |  |  | 0.015 |  |  |  |  |  | 0.086 |  |
|  | Control over time off | -0.160 | -0.389 | 0.068 | -0.097 |  |  | -0.405 | **-0.693** | **-0.117** | -0.281 |  |  |
|  | Control over daily hours | 0.030 | -0.163 | 0.224 | 0.022 |  |  | -0.004 | -0.234 | 0.227 | -0.003 |  |  |
|  | Overtime hours | 0.020 | -0.006 | 0.045 | 0.084 |  |  | 0.011 | -0.021 | 0.042 | 0.046 |  |  |
| **Model 3** | |  |  |  |  | 0.056 |  |  |  |  |  | 0.171 |  |
|  | Control over time off | -0.139 | -0.368 | 0.089 | -0.084 |  |  | -0.488 | **-0.776** | **-0.200** | -0.341 |  |  |
|  | Control over daily hours | 0.030 | -0.170 | 0.230 | 0.022 |  |  | 0.078 | -0.148 | 0.304 | 0.068 |  |  |
|  | Overtime hours | 0.023 | -0.003 | 0.048 | 0.097 |  |  | 0.017 | -0.013 | 0.047 | 0.073 |  |  |
|  | No children living at home | 0.004 | -0.274 | 0.281 | 0.001 |  |  | -0.502 | **-0.832** | **-0.171** | -0.202 |  |  |
|  | Married/co-habiting | -0.538 | **-0.964** | **-0.112** | -0.138 |  |  | -0.365 | **-0.718** | **-0.013** | -0.131 |  |  |
|  | Age | 0.018 | 0.004 | 0.033 | 0.146 |  |  | 0.022 | **0.003** | **0.040** | 0.153 |  |  |
|  | Lower skill level | -0.015 | -0.634 | 0.603 | -0.003 |  |  | 0.299 | -0.307 | 0.905 | 0.062 |  |  |
|  | Part-time work | -0.004 | -0.547 | 0.540 | -0.001 |  |  | -0.015 | -0.551 | 0.521 | -0.004 |  |  |
